# The effects of altered intrathoracic pressure on resting cerebral blood flow and its response to visual stimulation

**DOI:** 10.1016/j.neuroimage.2012.10.049

**Published:** 2013-02-01

**Authors:** Anja Hayen, Mari Herigstad, Michael Kelly, Thomas W. Okell, Kevin Murphy, Richard G. Wise, Kyle T.S. Pattinson

**Affiliations:** a Nuffield Division of Anaesthetics and Oxford Centre for Functional Magnetic Resonance Imaging of the Brain (FMRIB), Nuffield Department of Clinical Neurosciences, John Radcliffe Hospital, University of Oxford, Oxford, OX3 9DU, UK; b Cardiff University Brain Research Imaging Centre (CUBRIC), School of Psychology, Cardiff University, Park Place, CF10 3AT, Cardiff, UK

**Keywords:** Arterial spin labeling, Cerebral blood flow, Dyspnea, Functional magnetic resonance imaging, Respiratory loading, Transcranial Doppler sonography

## Abstract

Investigating how intrathoracic pressure changes affect cerebral blood flow (CBF) is important for a clear interpretation of neuroimaging data in patients with abnormal respiratory physiology, intensive care patients receiving mechanical ventilation and in research paradigms that manipulate intrathoracic pressure. Here, we investigated the effect of experimentally increased and decreased intrathoracic pressures upon CBF and the stimulus-evoked CBF response to visual stimulation.

Twenty healthy volunteers received intermittent inspiratory and expiratory loads (plus or minus 9 cmH_2_O for 270 s) and viewed an intermittent 2 Hz flashing checkerboard, while maintaining stable end-tidal CO_2_. CBF was recorded with transcranial Doppler sonography (TCD) and whole-brain pseudo-continuous arterial spin labeling magnetic resonance imaging (PCASL MRI).

Application of inspiratory loading (negative intrathoracic pressure) showed an increase in TCD-measured CBF of 4% and a PCASL-measured increase in grey matter CBF of 5%, but did not alter mean arterial pressure (MAP). Expiratory loading (positive intrathoracic pressure) did not alter CBF, while MAP increased by 3%. Neither loading condition altered the perfusion response to visual stimulation in the primary visual cortex. In both loading conditions localized CBF increases were observed in the somatosensory and motor cortices, and in the cerebellum.

Altered intrathoracic pressures, whether induced experimentally, therapeutically or through a disease process, have possible significant effects on CBF and should be considered as a potential systematic confound in the interpretation of perfusion-based neuroimaging data.

## Highlights

► We examined how alterations in intrathoracic pressure affect cerebral blood flow. ► Decreased intrathoracic pressure increased global cerebral blood flow. ► The perfusion response to visual stimulation remained unaffected. ► Localized perfusion increases were observed that related to respiratory work. ► Alterations in intrathoracic pressure may confound interpretation of FMRI.

## Introduction

The brain regulates its blood supply in order to ensure adequate delivery of oxygen and nutrients in the face of changing systemic blood pressure and regional changes in metabolic demand. Cerebral autoregulation is the hemodynamic process that keeps cerebral blood flow (CBF) within tight physiological limits during changes in perfusion pressure via adaptations in cerebrovascular resistance (van Beek et al., 2008), while neurovascular coupling matches local CBF to changes in underlying neuronal activity (Nielsen and Lauritzen, 2001).

CBF can be affected by physiological modulations or pharmacological agents, which in turn can affect the CBF-derived blood oxygen level dependent (BOLD) response (Brown et al., 2003; Cohen et al., 2002). Altered intrathoracic pressures could potentially affect CBF via direct pressure effects on the great arteries and veins of the chest, causing systemic changes in arterial blood pressure and in venous return from the brain (Kolbitsch et al., 2000).

Classical physiology teaching states that CBF is affected by systemic factors (e.g. systemic vascular resistance, arterial blood pressure, venous return, arterial carbon dioxide and oxygen tensions) and by local factors within the brain (e.g. autoregulation). It is therefore important to examine how these effects might confound the interpretation of CBF-derived functional neuroimaging methods, such as arterial spin labeling (ASL) or BOLD contrast, when intrathoracic pressures are changed. In order to correctly interpret FMRI to examine brain activity of patients with altered intrathoracic pressure or of healthy volunteers undergoing respiratory loading, we need to establish the effects of respiratory loading, both on baseline CBF and on CBF changes during neuronal activation. This understanding of the effects of altered intrathoracic pressure on CBF may be important in a number of clinical and experimental scenarios.

The ability to quantify CBF regulation in patients following neurological injury is commonly required in both research and clinical practice. As these patients can often require intensive care interventions such as sedation and mechanical ventilation, changes in intrathoracic pressure may potentially affect measurements of CBF. During mechanical ventilation, gas is forced into the lungs, increasing intrathoracic pressures to + 15 to + 30 cmH_2_O. Additionally, positive end expiratory pressure (PEEP) of up to + 10 cmH_2_O is often applied to prevent lung collapse. Any known effect of mechanical ventilation on CBF would need to be taken into consideration when interpreting CBF measures.

Changes in intrathoracic pressure might also be a potential confound when interpreting functional neuroimaging in patients with respiratory diseases. There is currently much interest in using FMRI to understand the relevance of brain processes in these often chronic and debilitating conditions. In COPD, there is fixed obstruction of airways and inspiratory pressure of approximately − 13 cmH_2_O, and expiratory pressures of + 3 cmH_2_O have been reported (Montes de Oca et al., 1996). Acute bronchoconstriction in asthma can generate negative intrathoracic pressures of − 30 cmH_2_O (Stalcup and Mellins, 1977). In addition to clinical asthma, methacholine, a compound that is inhaled to create bronchoconstriction, is used to test for bronchial hyperreactivity or model asthma for experimental purposes, and could conceivably be used to investigate brain mechanisms of asthma with FMRI.

Furthermore, intrathoracic pressure is altered by breathing against an external resistive respiratory loads as an integral part of laboratory models used to investigate the neuronal mechanisms involved in the control of breathing and in the perception of breathlessness, e.g. (Banzett et al., 2008; Sturdy et al., 2004). These models can be used to gain an understanding of the neuronal mechanisms involved in the perception of breathlessness with FMRI (Banzett et al., 2008; von Leupoldt et al., 2007). Although the profound CBF effects of the Valsalva maneuver (Tiecks et al., 1996) and Mueller maneuver (Reinhard et al., 2000) have been well described, these techniques, which involve forced expiration or inspiration against a closed glottis, are extreme, unphysiological, and induce other physiological processes beyond changing thoracic pressure (e.g. hypercapnea). Therefore, to better understand the effects of altered intrathoracic pressure on cerebral physiology, a controlled experimental paradigm is required.

In this study we examined the effects of inspiratory and expiratory loading on CBF measured in two ways, with ASL FMRI and with transcranial Doppler sonography (TCD). We also determined whether or not respiratory loading induces CBF changes that might alter the perfusion response to visual stimulation. Informed by previous studies using TCD during inspiratory loading (decreased inspiratory pressure), we hypothesize that inspiratory loading will lead to increased CBF and that expiratory loading (increased expiratory pressure) will lead to a decrease in CBF. If the induced CBF changes are relatively large, these could potentially alter the CBF response to visual stimulation, similar to the way in which CBF changes affect evoked BOLD responses (e.g. increased arterial CO_2_ increases CBF, which decreases the BOLD response to visual activation (Cohen et al., 2002)). We furthermore expect inspiratory and expiratory loading to show locally increased perfusion in brain areas that are engaged in the control of breathing.

## Materials and methods

### Brief overview

The study consisted of three sessions: a training session, a laboratory session in which CBF was measured indirectly (with TCD) with simultaneous continuous non-invasive mean arterial blood pressure (MAP) measurements and a third session in the MRI scanner during which CBF was measured across the whole brain with ASL FMRI.

TCD enables the beat-by-beat investigation of local blood velocity changes in the middle cerebral artery (MCAV), which reliably correlate with changes in CBF (Bishop et al., 1986) and hence can be used as an indirect measure of CBF, as long as a stable diameter of the insonated vessel and constant blood vessel resistance and permeability throughout the brain can be assumed.

ASL allows serial measurement of relative CBF to be taken over several minutes from the whole brain (Dai et al., 2008). During ASL, water molecules in flowing blood are magnetically labeled at the level of the neck. An image is acquired in the brain once this labeled blood water has exchanged from the vasculature into the brain tissue. Tissue perfusion, or CBF, can then be assessed by subtracting this labeled image from a control image in which the inflowing blood water is not magnetically labeled. We used a recently developed variant of ASL, PCASL (Dai et al., 2008), which provides enhanced labeling efficiency and a resultant increase in the signal-to-noise ratio of the CBF measurements.

### Participants

Twenty volunteers (mean age 27 (SD ± 6) years, 6 females) participated in this study after giving written informed consent in accordance with the Oxfordshire Research Ethics Committee. Participants were healthy right-handed non-smokers with no history of significant neurological, pulmonary or cardiovascular disease and free from acute illness (e.g. acute upper respiratory tract infection). Participants abstained from excessive consumption of alcohol (24 h), caffeine (6 h) and heavy meals (2 h) before the experiment and had sufficient sleep the preceding night. Female participants either used hormonal contraception or were at the same stage of their menstrual cycle during both parts of the experiment.

The 20 volunteers were drawn from a total of 27 participants, seven of which were excluded for the following reasons: one did not tolerate the visual stimulus, four did not comply with the study protocol, and two displayed excessive head motion during FMRI. These complete datasets were excluded from all analyses.

### Respiratory loading

**Fig. 1:**
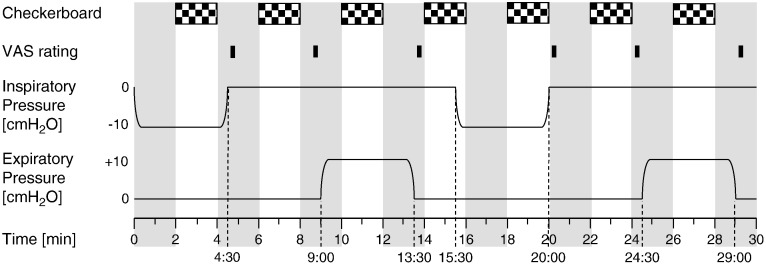
Timeline of experimental paradigm. A 4½-minute block of inspiratory resistance of − 9 cmH_2_O is followed by 4½ min of unloaded breathing at 4:30 min and 4½ min of expiratory resistance of + 9 cmH_2_O at 9:00 min. After a normal breathing period of 2 min at 13½ min, this is followed by a second cycle of inspiratory loading (4½ min from 15½ min onwards), unloaded breathing (4½ min, starting at 20:00) and expiratory resistance (4½ min from 24½ min onwards). 10 s after each resistance block and 10 s before the end of the unloaded breathing blocks, participants were presented with a VAS scale for 10 s to rate their average breathlessness over that block. A 2 Hz black and white flashing checkerboard was presented for 120 s every 240 s, starting at 2 min. PETCO_2_ values remained visible throughout baseline and visual stimulation.

**Fig. 2:**
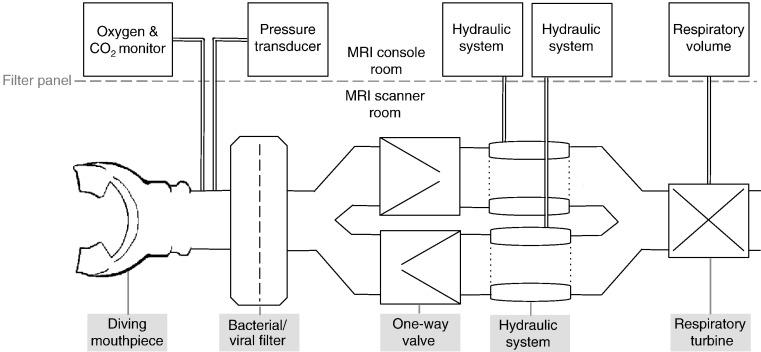
Schematic diagram of respiratory circuit. A diving mouthpiece (Scubapro UK Ltd, Mitcham, UK) connects to a bacterial and viral filter (Vitalograph, Buckingham, UK) from which respiratory gases and respiratory pressure are sampled via polyethylene extension tubing (Vygon SA, Ecouen, France). One sampling line leads to a pressure transducer (MP 45, ± 50 cmH_2_O, Validyne Corp., Northridge, CA, USA) connected to an amplifier (Pressure transducer indicator, PK Morgan Ltd, Kent, UK). During the physiology session, the second sampling line connects to a gas analyzer that samples oxygen and CO_2_ (Capnomac Ultima, Datex Ohmeda, Helsinki, Finland), while during the scanning session tidal CO_2_ was measured with a rapidly responding gas analyzer (CD-3A and S-3A; AEI Technologies, Pittsburgh, PA, USA). The filter connects to a Y-piece, creating an inspiratory and an expiratory limb through one-way valves (Hans Rudolf, Kansas City, MO, USA). The diameter of a 5 cm long section of the inspiratory and the expiratory channel can be altered remotely via a hydraulic system. Each modifiable section of tubing is lined with a rubber party balloon connected via non-distensible plastic tubing to a 20 ml syringe filled with water and used for inflation and deflation of the balloon. The inspiratory and expiratory tubes recombine through a Y-piece and an attached turbine (VMM-400, Interface Associates, Aliso Viejo, CA, USA) records inspiratory (V_i_) and expiratory (V_e_) volumes.

The same experimental paradigm was performed in both experimental sessions (Fig. 1). In a supine position, participants wore a nose clip (Air Safety Ltd, Morecambe, UK) and standard foam earplugs while breathing room air through a mouthpiece connected to a custom-built respiratory circuit (shown in Fig. 2). Participants were exposed to intermittent inspiratory or expiratory loads of either minus or plus 9 cmH_2_O, which was induced by partially occluding either the inspiratory or the expiratory limb of the breathing circuit with a balloon that was remotely controlled by a hydraulic system. Intrathoracic pressure can be inferred through non-invasive measurements of mouth pressure, which is approximately equivalent to measurements of esophageal pressure, the common way to determine intrathoracic pressure (Baydur et al., 1982).

### Respiratory control

As a strong cerebrovascular vasodilator, CO_2_ has been shown to alter CBF and to induce changes in the BOLD FMRI signal (Cohen et al., 2002). Therefore, participants were trained to maintain the partial pressure of end-tidal CO_2_ (PETCO_2_) stable throughout this study. A PETCO_2_ baseline value was determined for each individual at the beginning of each experimental session by monitoring PETCO_2_ over two minutes after an 8-minute habituation period. A PETCO_2_ target value was set 0.1 kPa lower than the baseline. During the training session, participants learnt to keep their PETCO_2_ stable. At the beginning of each session, participants were instructed to keep their PETCO_2_ stable within 1 kPa of their target value throughout the paradigm via numerical visual feedback that was back-projected to participants onto the centre of a screen behind them, visible via a mirror (similar set-up to Pattinson et al. (2007)).

All physiological data were sampled at 50 Hz and were logged via a Power1401 (CED, Cambridge, UK) using Spike2.7 (CED, Cambridge, UK). PETCO_2_ was calculated on a breath-by-breath basis through an online script in Spike2.7 and was fed back to the participant via a stimulus presentation program (Presentation, Neurobehavioral Systems, Albany, USA) on a second computer.

Behavioral responses were made using a custom-made button box and were represented on a horizontal visual analog scale (VAS). This scale allowed participants to answer the question “How strong was your breathlessness averaged over the last 4½ min block?” with the anchors ‘none’ on the left and ‘unbearable’ on the right. Participants were told to interpret the bar as a scale from 0 to 10, where 9 would indicate severe breathlessness and the wish to terminate the experiment.

Every 4 min, participants were shown a 2 Hz flashing checkerboard for 2 min (see Fig. 1). A 2 Hz flashing checkerboard induces robust activations of the visual cortex, hence allowing us to detect potential intrathoracic pressure related changes in the perfusion increase to visual activation with high sensitivity.

The numerical PETCO_2_ feedback was continuously displayed during the whole experimental period in a small box with a black background displayed in the centre of the screen. The only time PETCO_2_ feedback was not shown, was whilst participants gave subjective ratings on a VAS-scale (six periods of ten seconds).

### Recordings during laboratory session

A 2.0 MHz pulsed wave Doppler transducer (DWL Elektronische Systeme GMbH, Sipplingen, Germany), held over the right temporal bone window, provided continuous MCAV. Standard vessel identification criteria were used and the signal was sampled at the individual's optimum depth between 50 and 55 mm. Continuous non-invasive blood pressure was measured with a Finapres blood pressure monitor (Ohmeda 2300, BOC Health Care, USA). The cuff was applied to the midphalanx of the middle or ring finger of the non-dominant hand and placed at heart level. Beat-to-beat systolic and diastolic pressures, as well as MAP, were computed. MAP was calculated as one-third of systolic blood pressure (SBP) plus two-thirds of diastolic blood pressure (DBP). Pulse pressure (PP) was calculated as the difference between SBP DBP. Cerebrovascular resistance (CVR) was calculated with the formula CVR = MAP / MCAV. Blood pressure was recorded during the laboratory session only, due to the lack of availability of an MRI compatible device. Heart rate was measured with a pulse oximeter (Capnomac Ultima, Datex Ohmeda, Helsinki, Finland). Respiratory measures were obtained as explained in Fig. 2.

### Recordings during MRI session

Participants were scanned with a 3 tesla Siemens Verio MRI scanner (Siemens, Erlangen, Germany) with a 32-channel radiofrequency receive coil, using foam inserts to minimize head motion. A whole-brain PCASL sequence was utilized (Dai et al., 2008) with a gradient-echo echo-planar (EPI) readout (PCASL module flip angle 20°, labeling duration = 1.4 s, post labeling delay = 1 s, EPI readout flip angle 90°, TR = 4.0 s, TE = 14 ms, 6/8 k-space). We acquired twenty-two axial slices in ascending order (450 volumes, 3.8 × 3.8 × 5.0 mm voxels, interslice gap = 1.75 mm,). Pre-saturation of the imaging region was performed just prior to labeling to suppress static tissue signals, reducing the sensitivity to subject motion and physiological noise. A time-of-flight angiography scan was acquired in the neck to allow visualization of the main feeding arteries. The PCASL labeling plane was positioned approximately halfway between the upper and lower contortions of the vertebral arteries, so that the plane was perpendicular to both the vertebral and carotid arteries, thereby optimizing the labeling efficiency. For each subject, an MPRAGE structural image was acquired to enable registration of functional data. Continuous heart rate was recorded with a pulse oximeter (MR multigas monitor, MR Equipment, model 9500) and respiratory measures were obtained as described in Fig. 2.

### Data analysis

#### Physiological recordings

Data were exported to MatLab (MathWorks Inc., Natick, MA, USA) and analyzed using custom-written scripts. Respiratory parameters (CO_2_ partial pressures and respiratory pressures), circulatory parameters (heart rate and MAP) and MCAV were averaged within subject across each of the six respiratory resistance periods (unloaded breathing, inspiratory loading and expiratory loading [× 2]). No difference between the first and the second repeat of a condition were found and the results for both repeats were combined.

#### PCASL

Analysis of the PCASL FMRI images to identify regions exhibiting significant perfusion changes was carried out using tools from the FMRIB Software Library (FSL, www.fmrib.ox.ac.uk/fsl). Full perfusion signal modeling was performed in FEAT (FMRI Expert Analysis Tool in FSL), with the ASL tag-control difference and the stimulus time courses modeled as separate explanatory variables (EV) and perfusion change obtained from their interaction. Non-brain structures (i.e. skull and surrounding tissues) were removed using BET (Smith, 2002) and 4D images were motion corrected using MCFLIRT (Jenkinson et al., 2002). Spatial smoothing using a Gaussian kernel of full-width-half-maximum 5 mm and high pass temporal filtering were performed (Gaussian-weighted least-squares straight line fitting with a high-pass-filter cutoff of 12 s). After preprocessing, the functional scans were co-registered to the individual's structural scan (linear registration) and then to the MNI152 standard space (average T1 brain image constructed from 152 normal subjects at the Montreal Neurological institute, Montreal, QC, Canada) using nonlinear registration. ASL images were corrected for physiological noise using RETROICOR (Glover et al., 2000; Harvey et al., 2008). RETROICOR involves fitting low-order Fourier series to the EPI data based on the time of each image acquisition relative to the phase of the cardiac (3 terms) and respiratory cycles (4 terms) and their interaction (1 term).

FEAT (http://www.fmrib.ox.ac.uk/fsl/feat5/index.html) was used to compare the relative CBF change in response to inspiratory and expiratory resistance to CBF while breathing was unloaded. The general linear model (GLM) included the following EVs: Mouth pressure during inspiration (P_min_), mouth pressure during expiration (P_max_), PETCO_2_, visual stimulation, the act of rating, breathlessness score and six motion parameters. Interactions between inspiratory loading and visual activation and expiratory loading and visual activation were modeled. On the second level (group level), FEAT was used to perform a voxel-wise statistical analysis using a mixed effects model to combine the GLM results of individual participants while accounting for intersubject variance. A cluster threshold of Z > 2.3 and a (corrected) cluster significance threshold of P = 0.05 were used.

An anatomical mask of the primary visual cortex (V1) was created using the Juelich histological atlas within FSLView (http://www.fmrib.ox.ac.uk/fsl/fslview/index.html) with a probability threshold applied at 0.35. This mask was used to determine perfusion changes in the visual cortex in response to visual stimulation and the interactions between visual stimulation and respiratory loading.

A region of interest approach was used to calculate mean fractional perfusion changes in response to inspiratory and expiratory loading in the whole brain grey matter (grey matter masks determined individually by segmenting participants' structural scans with FAST (FMRIB's Automated Segmentation Tool, http://www.fmrib.ox.ac.uk/analysis/research/fast/)), and in the individual brain areas with significantly increased perfusion demonstrated in the voxel-wise analysis.

It is possible that localized CBF increases (e.g. activation due to the work of breathing against load etc.) might elevate the general global CBF change. It is also possible that a small collinearity between respiratory loading and the visual stimulation could inflate the global grey matter increase. Therefore, to account for both of these potential effects, we performed a second region of interest analysis specifically excluding localized CBF increases (i.e. sensorimotor cortices and cerebellum) that were assumed to be task specific and also excluding V1.

In order to compare our results to the well-known effects of CO_2_ on cerebral perfusion (Cohen et al., 2002), we included the natural fluctuations in PETCO_2_ in the statistical model (i.e. although participants attempted to keep PETCO_2_ constant, spontaneous fluctuations and small imperfections in performing this task were present (Wise et al., 2004)).

To retrospectively evaluate the sensitivity of our study design, we used the study sample size and the standard deviations from our region of interest analyses to statistically determine the smallest grey matter and V1 perfusion increases that our study is able to detect with 80% confidence.

## Results

### Laboratory session

**Fig. 3:**
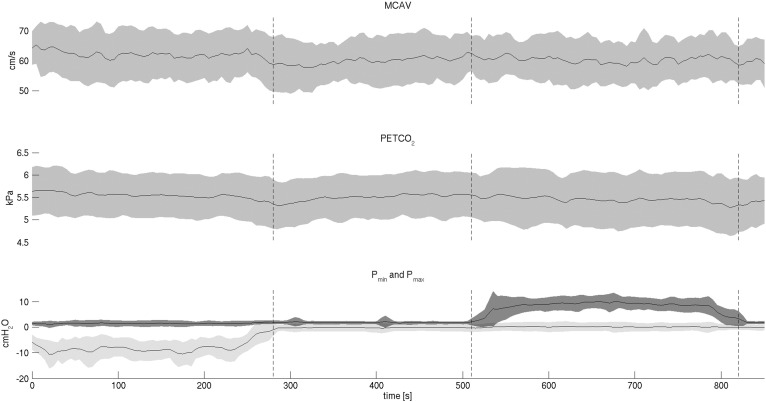
Middle cerebral artery velocity (MCAV), partial pressure of end-tidal CO_2_ (PETCO_2_), minimum pressure at the mouth (Pmin, light grey SD) and maximum pressure at the mouth (Pmax, dark grey SD) averaged across 20 participants and plotted over the first half of the physiological recording session (mean ± SD).

Physiological results of the laboratory session are reported in the top part of Table 1. There was no significant difference between the first and second block of each stimulation condition, hence values are presented as averages over both blocks. Mean arterial pressure increased from 90.7 ± 15.2 mm Hg to 93.8 ± 15.7 mm Hg (p < 0.01), during expiratory loading, while PETCO_2_ and HR remained stable for both loading conditions. The time courses of MCAV, PETCO_2_ and minimum and maximum mouth pressure values throughout the first half of the laboratory session are illustrated in Fig. 3.

### MRI session

**Fig. 4:**
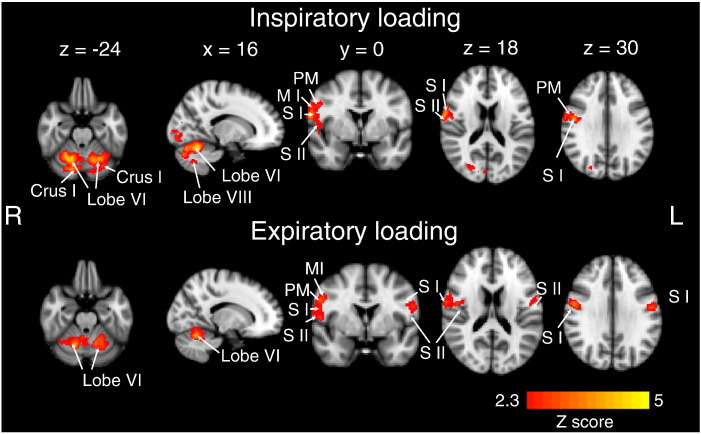
Significant CBF increases during inspiratory and expiratory loading compared to unloaded breathing. The images consist of a colour-rendered statistical map superimposed upon a standard (MNI) brain. Significant regions are displayed with a threshold of Z > 2.3 and a cluster probability threshold of p < 0.05 (corrected for multiple comparisons). PM = premotor area, M1 = motor cortex, S I = primary somatosensory cortex, S II = secondary somatosensory cortex.

The mean changes in relative CBF (± SD) in the grey matter and in V1 are reported in Table 2. Areas in which perfusion was increased during inspiratory loading are shown in the top panel of Fig. 4. Areas in which perfusion was increased during expiratory loading are shown in the bottom panel of Fig. 4. Locations of peak perfusion increase, Z scores of peak voxels (Z_max_) and % perfusion increase during inspiratory and expiratory loading within the respective activity masks are provided in Table 3. No relative CBF decreases were seen in any of these regions of interest during the different loading conditions.

In response to inspiratory loading, significant regional increases in perfusion were found in the right primary and secondary somatosensory cortices, the right premotor and primary motor cortices and in cerebellar lobe VI. Significant perfusion increases in the somatomotor cluster extended over the primary and secondary somatosensory cortices and the primary motor cortex and the premotor cortex. The locations and Z scores (Z_max_) of local peak voxels are shown in Table 3. Additional areas commonly activated in respiratory studies using BOLD showed increased perfusion in our data only when no cluster threshold was applied. Table 4 shows the location of peak voxels in clusters that showed a significant Z score of above 2.3, but that did not survive the application of the cluster threshold.

Even when the localized brain activity related to increased breathing effort and V1 was excluded from analysis, global CBF remained increased during inspiratory loading, which indicates a small, but significant increase in baseline CBF (+ 0.5% per cmH_2_O, p < .05, Table 2).

During application of expiratory loading, relative CBF was increased in the bilateral somatosensory and motor cortices and in the cerebellar lobe VI. The locations of signal maxima and Z scores are shown in Table 3.

The voxel-wise analysis demonstrated a robust bilateral increase in CBF in the visual cortex in response to visual stimulation (Z_max_ = 7.11, not shown). We calculated a 14.7% increase in relative in CBF response to visual stimulation compared to baseline in a standard anatomic mask of the V1. No significant interactions were observed between the relative CBF response to visual stimulation and inspiratory or expiratory loading (Table 2).

Results of the physiological recordings during the scanning session are reported in the lower part of Table 1 and show stable PETCO_2_ and HR for all respiratory conditions. As during the laboratory session, there were no differences between the first and second block of each condition, hence values are presented as averages over both blocks.

Retrospective estimation of the power of the study based on sample size and standard deviations from the region of interest analysis demonstrated that the study can detect a perfusion change of 0.5% of global grey matter perfusion with 80% confidence. A change of 1.3% of the perfusion increase in response to visual stimulation can be detected with 80% confidence to determine possible interactions between visual stimulation and inspiratory or expiratory resistance in V1.

## Discussion

### Summary

The key findings of this study are:

1) Inspiratory loading caused an increase in relative CBF of approximately 0.5% per cmH_2_O load pressure, but did not have any effect upon arterial blood pressure. This global increase in relative CBF remained evident even when localized CBF increases in brain areas thought to be responsible for performing the breathing tasks and the primary visual cortex were excluded in a subsequent region of interest analysis.
2) Expiratory loading had no effect on global CBF (either including or excluding task-specific regions of interest and the primary visual cortex), but was associated with an increase in mean arterial pressure from 90.7 ± 15.2 mm Hg to 93.8 ± 15.7 mm Hg (approximately 0.3% per cmH_2_O, p < 0.01).
3) There was no generalized modulation of the CBF response to visual stimulation in the primary visual cortex by either inspiratory or expiratory resistive loading.
4) Localized perfusion increases were observed in the somatosensory and motor cortices and the cerebellum during inspiratory and expiratory loading.

### CBF changes with inspiratory and expiratory loading

This study was motivated by the need to understand how the well-described effects of respiratory loading on systemic arterial and venous systems (Scharf, 1991) might affect CBF. Other studies of respiratory loading employing a variety of different techniques have shown varying effects on CBF (Cooke et al., 2006; Droste et al., 1999; Kolbitsch et al., 2000; Lorenz et al., 2001; Reinhard et al., 2000). The conflicting results of previous studies may possibly depend on the specific methods used to generate respiratory loads, the differing techniques used to measure CBF and the fact that studies tended to only apply either inspiratory or expiratory loading and the effects might therefore not be directly comparable. With ASL, we have additionally been able to examine the effect of regional changes in CBF across the whole brain and to test how the CBF response to neural activation might be affected by respiratory loading.

### Arterial blood pressure and CBF

Inspiratory loading elicited no changes in blood pressure (although CBF increased), while expiratory loading caused a small increase in blood pressure (but was not associated with altered CBF). As both experimental sessions were otherwise performed in an identical manner, and as the physiology measurements and CBF changes in both sessions were comparable, we assume that similar blood pressure changes occurred during the MRI scanning session. In terms of understanding CBF regulation, our data therefore suggest that small changes in blood pressure are directly caused by expiratory loading, but that a different mechanism is responsible for the increase in CBF observed during inspiratory loading. We speculate that the increase in blood pressure during expiratory loading might occur due to a primary localized direct pressure effect of increased intrathoracic pressure on the great arteries of the chest, e.g. the aorta, as other cardiovascular parameters (heart rate, pulse pressure) were unchanged, making it unlikely that the increased blood pressure was secondary to systemic factors, such as increased sympathetic activation or increased systemic vascular resistance.

### Venous return

Venous return from the brain to the heart is important for the regulation of the cerebral circulation. In an upright position, gravity maintains a slightly negative pressure in the jugular veins, resulting in unimpeded venous drainage from the brain. Venous drainage is further assisted by the “thoracic pump”, the slight negative intrathoracic pressure generated during normal inspiration that is transmitted to the great veins in the thorax (e.g. the superior vena cava). In a supine position like in this study, gravity no longer has such a profound effect and jugular venous pressure (JVP) increases to approximately 8 mm Hg (Leonard et al., 2008), potentially impeding venous outflow from the brain. Decreased intrathoracic pressure caused by inspiratory loading in a supine position decreases JVP through direct pressure effects on the great veins in the thorax that are in direct continuity with the jugular veins. Inspiratory loading therefore may act to accentuate the normal thoracic pump mechanism and will therefore increase the arterio-venous pressure gradient across the brain, leading to increased CBF.

Increased intrathoracic pressure during expiratory loading has the opposite effect on the central venous circulation and will tend to elevate the pressure in the jugular vein, decreasing the arterio-venous pressure gradient across the brain. Without an effective compensatory mechanism, this would decrease CBF. We therefore speculate that this reduction in venous return would be compensated by the increased MAP during expiratory loading, leading to unchanged CBF. Jugular venous pressure was not measured during this study (due to the invasive nature of this measurement), but the effects of intrathoracic pressure on JVP have been well documented (Hall and Guyton, 2011).

### Systemic vascular resistance

Another possibility is that alterations in atrial transmural pressure induced by respiratory loading stimulate the release of atrial natriuretic peptide, a powerful vasodilator that decreases systemic vascular resistance. The difference between the systolic and diastolic blood pressures (i.e. pulse pressure) is proportional to systemic vascular resistance. Although we observed an increase in mean arterial pressure during expiratory loading, this was associated with equivalent increases in diastolic and systolic pressures, while pulse pressure remained constant. Therefore, the increase in CBF during inspiratory loading is unlikely to originate from altered systemic arterial tone and we therefore conclude that alterations in venous return may explain the increased CBF during inspiratory loading.

### Arterial CO_2_

Additionally, arterial CO_2_ tension has a strong impact on the regulation of CBF. For each 1 mm Hg change in PaCO_2_, there is a corresponding CBF change in the same direction of approximately 2.5% in hypocapnia, and 4.5% in hypercapnia (Ide et al., 2003) and there are correspondingly profound effects on the BOLD response, which is decreased in hypercapnia and increased in hypocapnia. As changes in respiration potentially affect PaCO_2_ (and therefore CBF), we have paid meticulous attention to the maintenance of constant CO_2_ levels. We trained our research participants to maintain their PETCO_2_ constant through a visual feedback system. Without this training, respiratory loading, equivalent to that used here during spontaneous breathing, is well documented to cause hypercapnia due to decreases in ventilation (Davies et al., 1919), of between 1 and 3 mm Hg, therefore further increasing CBF by approximately 4.5% to 13.5%. Such a CBF increase would confound the interpretation of our results. As we have maintained constant PETCO_2_, we can be confident that the CBF increase during inspiratory loading is a true primary physiological effect, that taken in isolation is small, but could contribute to a more profound systematic confound due to secondary effects of loading on ventilation.

Such careful control of CO_2_ levels is important not only in respiratory studies, but also in any other paradigm that may alter respiration. One example of the importance of respiratory confounds is the caffeine-induced decrease of CBF (Chen and Parrish, 2009), which confounds the BOLD response. Caffeine stimulates both the respiratory system (causing hypocapnia (Bell et al., 1999)) and the cardiovascular system (increasing blood pressure (Robertson et al., 1978)), both important contributors to CBF regulation. We are not aware of any current reports of caffeine's effect on CBF having obtained what we consider essential measures of systemic physiology and its direct effects on CBF are therefore not clear.

### Interactions between respiratory loading and visual stimulation

Inspiratory and expiratory loading did not modulate the stimulus-evoked perfusion increase in V1. Therefore, the theoretical confounding effects of ABP and of baseline CBF on stimulus-evoked CBF responses as suggested by Payne (2006) were not observed. This finding is in agreement with a recent paper by Gauthier et al. (2011), demonstrating a profound effect of carbogen on baseline CBF and the visually-evoked BOLD response in the visual cortex during simultaneous carbogen inhalation and visual stimulation, which were evident at the lowest level of carbogen inhalation (2.5% CO2, 38.25% O2, balance N_2_), while relative CBF increases in the visual cortex induced by visual stimulation remained unaffected during carbogen inhalation.

The interpretation of the BOLD signal, which is a composite signal comprising CBF, cerebral blood volume and the cerebral metabolic rate of oxygen (Kim et al., 1999), is highly susceptible to changes in blood deoxyhemoglobin level and to the size of the perfusion response to activation (Gauthier et al., 2011). A small CBF increase, as seen during inspiratory loading in our study, could hence lead to a lower fraction of deoxyhemoglobin, an elevated BOLD baseline and a reduced BOLD response to functional tasks. Pooling of venous blood due to increased venous pressure might lead to a further decrease in the BOLD response (Lorenz et al., 2001). As such, CBF effects might confound the comparison of BOLD activations across different levels of inspiratory loading, e.g. in von Leupoldt et al. (2009), where the CO_2_ effects of loading will affect CBF in addition to the direct effects of negative intrathoracic pressure. Similarly, Evans et al. (1999) compared positive pressure ventilation with spontaneous breathing, hence their results might be confounded due to opposing effects on CBF and venous drainage.

As we only used ASL during this study, we can only comment on the CBF component of the BOLD signal. One approach to control for potential CBF influences on the BOLD response is to acquire a measure of baseline CBF, which can be used as a regressor to aid the differentiation of local metabolic effects from purely vascular effects when interpreting BOLD signal change (Pattinson et al., 2009). Intrathoracic pressure changes might additionally affect other components of the BOLD signal, which could be investigated in future studies.

### Implications for the understanding of respiratory control

Activation in somatosensory, motor and cerebellar areas has previously been found to be implicated in neuroimaging studies of modified respiration (reviewed in Herigstad et al. (2011)). Increased perfusion in the premotor, motor and supplementary motor cortices is likely to indicate stronger mechanical breathing effort during loading periods, as these areas are implicated in conscious breathing (Pattinson et al., 2007, 2009). In the primary motor cortex, the location of the peak perfusion increase during expiratory loading was near to locations of peak activation in the orofacial region close to areas previously identified to activate in the performance of non-speech tasks involving the lips and tongue (reviewed by Takai et al. (2010)). While respiration is classically located in the trunk area of the homunculus (Penfield and Rasmussen, 1950; Penfield and Roberts, 1959), respiratory activation is more complex. A second, more ventrally located peak has been identified that shows the location of the larynx to overlap with the location of the expiratory muscles (Loucks et al., 2007). Inspiratory and expiratory loading in our study activate within this orofacial area, indicating the strong activation of respiratory muscles during these manipulations.

In the somatosensory cortex, inspiratory and expiratory loading each activated a distinct peak in the inferior precentral gyrus. Activation relating to inspiratory loading was unilateral (right side of the brain). On the right side the peak activations were spatially separated, although relatively close to each other (inspiratory loading: x = 62, y = 0, z = 18, expiratory loading: x = 54, y = − 4, z = 32,). These perfusion maxima are in a similar position to those identified during voluntary hyperpnea (McKay et al., 2003). However, these activation peaks are located considerably more ventrally in the somatosensory cortex when compared with the location of peak sites of activation in neuroimaging studies that manipulated respiration without employing a paced voluntary breathing task (Evans et al., 1999; von Leupoldt et al., 2008), indicating the importance of exact task specifications when placing respiratory-related perceptions in the somatosensory cortex. Our findings suggest that different aspects of respiratory sensation are processed within distinct areas within the somatosensory cortex, and that a more detailed study of this structure in relation to respiratory control would be worthwhile.

We have also shown increased perfusion in cerebellar lobe VI, which has previously been shown during resistive loading (Isaev et al., 2002), but its role in respiratory control is unclear. Based on increased BOLD signal in cerebellar lobe VI found during other sensorimotor tasks and during the application of aversive stimuli in other studies (Moulton et al., 2011), we speculate that this activity might be due to the sensorimotor control of breathing (sensorimotor cerebellum) and might also represent the negative psychological affect experienced during respiratory loading.

CBF increases that did not survive cluster thresholding were identified in the insula, amygdala and the anterior and posterior cingulate gyri in response to both inspiratory and expiratory loading. These brain areas have consistently been identified in neuroimaging studies using resistive loading to induce breathlessness (reviewed in Herigstad et al. 2011). It may be that the poorer signal to noise ratio of ASL explains why these CBF increases have not reached our statistical thresholds or that our experimental paradigm did not induce sufficient breathlessness to activate these structures. Furthermore, in a study by von Leupoldt et al. (2008) data analysis necessitated small volume correction to identify respiratory-related activity in these structures. We have chosen not to follow this analysis approach, as identifying respiratory-related activity in these structures was not a primary aim of this study.

### Clinical implications

The increase in CBF of ~ 5% caused by an inspiratory load of − 10 cmH_2_O has potential implications in the interpretation of functional neuroimaging data in a variety of clinical and experimental settings.

Negative intrathoracic pressure is the hallmark of respiratory obstruction in asthma and COPD. Neuroimaging in these conditions might be aimed at understanding how the brain contributes to respiratory obstruction (e.g. in asthma, higher cortical centres mediate increased activity in the vagus nerve, which innervates the bronchial musculature (Rosenkranz et al., 2005)) and to the perception of breathlessness (Banzett et al., 2008; Herigstad et al., 2011). Furthermore, experimental manipulations of the respiratory system in these patients might exacerbate negative intrathoracic pressures, e.g. by the administration of methacholine challenges in the investigation of asthma (Birnbaum and Barreiro, 2007), or by use of external respiratory loading to investigate breathlessness in both asthma and COPD.

Therefore, care should be taken when measuring and interpreting cerebral perfusion in patients with altered respiratory physiology. The small CBF increase caused by inspiratory loading might add to a change in underlying baseline physiology and could potentially alter FMRI results. Experimental interventions might affect CBF in other ways (e.g. by an effect on PETCO_2_ which was carefully controlled in the present study) that also need careful consideration. In these instances, care should be taken to include physiological monitoring (e.g. of PETCO_2_) and awareness and if possible measurements of potentially altered respiratory pressures during imaging analysis.

In the present study, although expiratory loading of + 10 cmH_2_O increased mean arterial blood pressure by a small amount, it did not significantly alter CBF, suggesting that at this level of loading autoregulation effectively compensates. Therefore, interventions such as CPAP (usually up to + 10 cmH_2_O) and the slightly raised expiratory intrathoracic pressure in COPD (+ 3 cmH_2_O) are unlikely to be important confounds in CBF interpretation. Mechanical ventilation (e.g. in intensive care patients) generates higher positive thoracic pressures (e.g. up to + 30 cmH_2_O, but usually + 15 to + 25 cmH_2_O) and it is conceivable that a greater load might affect CBF. In intensive care patients with brain injury (e.g. subarachnoid hemorrhage, stroke or traumatic brain injury), autoregulation might be impaired (Rowland et al., 2012) and it is therefore possible that small increases in blood pressure caused by mechanical ventilation will be translated directly into CBF increases. Adjustments of ventilator settings over time might additionally confound comparisons of CBF within those patients. It should also be considered that expiratory loading theoretically affects venous return and may have independent effects on BOLD responsiveness irrespective of CBF. It is furthermore worth noting that whilst stimulus-evoked ASL responses appear to be relatively robust in the face of baseline CBF changes, BOLD is more sensitive to such alterations in baseline CBF (Brown et al., 2003).

Research involving altered respiration can also benefit from using ASL in combination with careful PETCO_2_ control. The advantage of using ASL to detect respiratory-related CBF responses is that ASL gives higher temporal signal stability during longer stimulus blocks than BOLD FMRI (Wang et al., 2003), while not requiring the radioactive isotopes necessary for positron emission tomography (PET). The advantage of longer stimulus blocks is that longer periods of modulated breathing are more realistic with respect to patient experiences, while experimental respiratory sensations take some time to build-up (Evans et al., 2002). Furthermore, ASL allows the investigation of a single physiological parameter (i.e. CBF) as opposed to BOLD, which is a compounded signal encompassing different underlying physiological components, and which can therefore be influenced by physiological changes beyond CBF.

## Conclusion

Inspiratory loading at 9 cmH_2_O increases CBF by ~ 5%. We have demonstrated that this does not affect the ASL perfusion contrast, but we cannot exclude a systemic vascular effect that could potentially dampen the BOLD response in patients with clinical conditions involving decreased intrathoracic pressures. It appears that expiratory loading is better compensated for, as it does not change CBF when autoregulation is intact. Taking into account direct effects of intrathoracic pressure, especially during inspiratory loading, will allow the comparison of patients with altered respiratory physiology to healthy controls. Understanding how physiological factors may affect CBF in diseases such as COPD will level the path towards understanding disease mechanisms and drug action in the brains of patients with altered cerebrovascular and respiratory physiology.

## Disclosure/Conflict of interest

All authors declared that they have no conflicts of interest.

## Figures and Tables

**Table 1:** The top part of the table shows physiological measures during the laboratory session during unloaded breathing, inspiratory loading and expiratory loading. The lower part of the table shows physiological measures collected during the scanning session.

	Unloaded breathing	Inspiratory loading	Expiratory loading
*Physiology session*			
MCAV [cm/s]	60.6 ± 6.2	62.6 ± 7.0*	60.7 ± 6.1
CVR [mmHg/cm/s]	1.5 ± .30	1.4 ± .30**	1.53 ± .31
MAP [mmHg]	90.7 ± 15.2	89.8 ± 15.4	93.8 ± 15.7**
SBP [mmHg]	134.8 ± 19.1	134.1 ± 18.6	138.4 ± 17.9**
DBP [mmHg]	68.7 ± 14.6	67.8 ± 14.6	71.4 ± 16.0*
Pulse Pressure [mmHg]	66.1 ± 11.6	66.2 ± 9.8	67.0 ± 11.8
HR [bpm]	59.0 ± 7.3	59.3 ± 6.6	59.0 ± 6.7
Minute ventilation [l/min]	6.3 ± 2.0	5.9 ± 2.8	6.3 ± 2.0
Resp. Rate [breaths/min]	18.2 ± 11.5	16.3 ± 11.3	15.9 ± 10.0
PETO_2_ [kPa]	14.7 ± 0.8	14.6 ± 0.8	14.7 ± 1.1
PETCO_2_ [kPa]	5.5 ± 0.5	5.5 ± 0.5	5.5 ± 0.6
P_min_ [cmH_2_O]	− 0.1 ± 1.5	− 8.8 ± 2.6***	0.2 ± 1.7
P_max_ [cmH_2_O]	2.0 ± 0.7	1.6 ± 1.1*	9.3 ± 1.9***
Breathlessness [VAS 0-10]	0.9 ± 1.2	4.1 ± 1.6***	3.6 ± 2.0***

*MRI session*			
HR_(scan)_ [bpm]	55.9 ± 7.8	56.7 ± 7.3	56.6 ± 7.3
PETCO_2 (scan)_ [kPa]	41.3 ± 5.3	41.3 ± 5.3	41.3 ± 0.8
P_min (scan)_ [mmHg]	− 0.8 ± 0.4	− 9.8 ± 1.7***	0.2 ± 0.9**
P_max (scan)_ [mmHg]	1.0 ± 0.5	0.3 ± 0.9***	9.1 ± 2.0***
Breathlessness_(scan)_ [VAS 0-10]	0.7 ± 1.0	4.1 ± 1.9***	3.9 ± 2.1***

Stars indicate statistically significant differences between respective loading condition and unloaded breathing. * p < .05, ** p < .01, *** p < .001. Mean ± SD MCAV (middle cerebral artery velocity), MAP (mean arterial pressure), SBP (systolic blood pressure), DBP (diastolic blood pressure), pulse pressure, CVR (cerebrovascular resistance), HR (heart rate), PETCO_2_ (partial pressure of end-tidal CO_2_), PETO_2_ (partial pressure of end-tidal oxygen), minute ventilation, resp. rate (respiratory rate), P_min_ (minimum pressure at mouth), P_max_ (maximum pressure at mouth) and breathlessness rating averaged over both 4½ min blocks for each respiratory loading condition and during unloaded breathing. _(scan)_ indicates that data was collected inside the MRI scanner.

**Table 2:** Mean ± SD relative CBF changes measured with arterial spin labeling as percentage change from baseline in the global grey matter (total GM), the mean grey matter CBF excluding areas specifically activated in response to the loading task (non-task GM), the mean grey matter excluding areas specifically activated in response to the loading task and excluding the primary visual cortex (V1; non-task GM–V1) and in an anatomical mask of V1 in response to inspiratory and expiratory loading, CO_2_ , visual stimulation and the interaction between visual stimulation and inspiratory or expiratory loading.

Condition	Brain region	CBF change
Inspiratory load	Total GM	+ 0.5 ± 0.8* [%/cmH_2_O]
	Non-task GM–V1	+ 0.5 ± 0.8* [%/cmH_2_O]
Expiratory load	Total GM	+ 0.3 ± 0.8 [%/cmH_2_O]
	Non-task GM–V1	+ 0.2 ± 0.8 [%/cmH_2_O]
CO_2_	Total GM	+ 24.0 ± 11.3*** [%/kPa]
Visual stimulation	V1	+ 14.7 ± 5.8*** [%]
Interaction: visual and inspiratory loading	V1	+ 0.1 ± 1.9 [%]
Interaction: visual and expiratory loading	V1	+ 0.6 ± 1.9 [%]

Stars indicate statistically significant differences between respective loading condition and unloaded breathing. * p < .05, ** p < .01, ***p < .001.

**Table 3:** Peak voxel locations, peak voxel Z scores (Z_max_) and % perfusion increase (averaged over cluster) in activation clusters during inspiratory and expiratory loading.

Condition	Region	Peak voxel location	Z_max_	Perfusion increase [%/cmH_2_O]
Inspiratory loading	Right somatosensory cortex	X = 62, Y = 0, Z = 18	4.13	1.3 ± 0.9***
X = 48, Y = − 4, Z = 30	3.96
X = 62, Y = − 4, Z = 30	3.26
X = 52, Y = − 2, Z = 10	3.13
	Cerebellum	X = 16, Y = − 58, Z = − 24	5.09	1.5 ± 1.0***
Expiratory loading	Primary motor cortex	Right: X = 54, Y = − 4, Z = 32	Right: 4.01	1.0 ± 0.9***
X = 50, Y = − 10, Z = 38	3.95
X = 50, Y = − 8, Z = 34	3.86
Left: X = − 50, Y = − 8, Z = 40	Left: 3.78
X = − 52, Y = − 8, Z = 40	3.63
X = − 44, Y = − 12, Z = 38	3.41
	Somatosensory cortex	Right: X = 62, Y = − 6, Z = 16	Right: 3.35
X = 44, Y = − 8, Z = 22	3.25
X = 56, Y = 0, Z = 10	3.23
Left: X = − 56, Y = − 8, Z = 26	Left: 3.53
X = − 58, Y = − 4, Z = 24	3.49
X = − 52, Y = − 6, Z = 20	3.12
	Cerebellum	X = 18, Y = − 60, Z = − 22	4.23	1.4 ± 1.3***

Stars indicate statistically significant differences between respective loading condition and unloaded breathing. ***p < .001. A cluster probability threshold of p < .05 was applied to correct for multiple comparisons.

**Table 4:** Z scores (Z_max_) of peak voxels and coordinates of clusters of relative CBF increase in regions previously implicated in respiratory studies that did not survive the application of our cluster threshold during inspiratory and expiratory loading (data with uncorrected Z stats).

Condition	Region	Peak voxel location	Z_max_
Inspiratory loading	Left somatosensory cortex	X = − 58, Y = − 6, Z = 36	3.78
	Amygdala	Right: X = 30, Y = 2, Z = − 16	Right: 2.3
Left: X = − 14, Y = − 4, Z = − 22	Left: 2.2
	Right anterior cingulate gyrus	X = 0, Y = − 12, Z = 42	2.2
	Left posterior cingulate gyrus	X = − 4,Y = − 4, Z = 4	2.4
	Right anterior insula	X = 40, Y = 80, Z = − 2	2.5
	Right posterior insula	X = 40, Y = − 6, Z = 2	2.4
Expiratory loading	Right posterior insula	X = 38, Y = − 14, Z = 6	2.7
	Amygdala	Right: X = 24, Y = − 12, Z = − 12	Right: 2.6
X = 30, Y = 2, Z = − 16	2.0
Left: X = − 14, Y = − 2, Z = − 18	Left: 2.3
	Right anterior cingulate gyrus	X = 12, Y = 40, Z = 2	2.2
	Posterior cingulate gyrus	Right: X = 6, Y = − 14, Z = 46	Right: 2.3
Left: X = − 2, Y = − 48, Z = 2	Left: 2.7
